# Influence of Dimethyl Sulfoxide Pretreatment on the Push-Out Bond Strength of Fiber Posts Using Total-Etch and Self-Etch Adhesives: An In Vitro Study

**DOI:** 10.7759/cureus.94696

**Published:** 2025-10-16

**Authors:** Faaiza Mubeen, Rupa Ashok, Chakravarthy Arumugam, Mathan Rajan

**Affiliations:** 1 Conservative Dentistry and Endodontics, Sri Ramachandra Institute of Higher Education and Research, Chennai, IND

**Keywords:** dentin pretreatment, dimethyl sulfoxide (dmso), push-out bond strength, self-etch adhesive system, total-etch adhesive system

## Abstract

Background and aim

Endodontically treated teeth often require post placement to reinforce structural integrity. Fiber posts are preferred over metallic ones due to their elastic modulus, which is closer to that of dentin. Dimethyl sulfoxide (DMSO), known for its tissue penetration and solvent properties, has recently been explored as a dentin pretreatment to enhance adhesive performance. This study aimed to evaluate the effect of DMSO pretreatment on the push-out bond strength of fiber posts luted with different adhesive protocols, including self-etch and total-etch systems.

Materials and methods

Forty extracted human teeth were standardized and subjected to root canal treatment. Following post space preparation, the teeth were randomly divided into four groups (n = 10) based on adhesive protocols: Group I, total-etch + DMSO + single-step, seventh-generation universal adhesive (Palfique, Tokuyama Dental Corp., Tokyo, Japan); Group II, total-etch + primer + DMSO + three-step, fourth-generation adhesive (FL Bond II System, Shofu Inc., Kyoto, Japan); Group III, total-etch + DMSO + primer + three-step, fourth-generation adhesive (FL Bond II System, Shofu Inc.); and Group IV, self-etch + DMSO + single-step, seventh-generation universal adhesive (Palfique, Tokuyama Dental Corp.). Fiber posts were cemented using Estecem Plus resin cement and light-cured. All specimens were sectioned and subjected to push-out bond strength testing using a universal testing machine. Data were analyzed using IBM SPSS Statistics for Windows, Version 25.0 (Released 2017; IBM Corp., Armonk, NY, USA). One-way ANOVA was used for intergroup comparisons, Tukey’s post hoc test for multiple pairwise comparisons, and repeated-measures ANOVA for intragroup comparisons. Statistical significance was set at p < 0.05.

Results

Group II (2.34 MPa) showed the highest mean push-out bond strength. Statistically significant differences were observed between Groups II (2.34 MPa) and IV (1.39 MPa), III (2.24 MPa) and IV (1.39 MPa), I (0.68 MPa) and II (2.34 MPa), and I (0.68 MPa) and III (2.24 MPa). No significant differences were found between Groups II (2.34 MPa) and III (2.24 MPa) or between Groups I (0.68 MPa) and IV (1.39 MPa).

Conclusions

The application of DMSO in combination with the FL Bond II System, either before or after primer application, significantly enhanced the bond strength of fiber posts. Universal adhesives showed lower bond strength regardless of the etching protocol, suggesting that DMSO pretreatment is more effective when used with multistep adhesive systems.

## Introduction

Endodontically treated teeth are at increased risk of biomechanical failure due to significant loss of tooth structure, necessitating prosthetic restoration [1]. A direct correlation exists between the remaining tooth structure and resistance to occlusal forces [2]. Therefore, timely placement of a restoration with cuspal coverage is crucial following root canal treatment [3]. The decision to place a post largely depends on the amount and quality of the residual tooth structure.

The introduction of fiber posts has marked a major advancement in restorative dentistry, offering a reliable alternative to metal posts (cast or prefabricated). Unlike metal posts, fiber posts possess a modulus of elasticity similar to that of dentin [4]. These prefabricated posts feature a resin matrix, typically composed of epoxy resin or its derivatives [5]. Fiber-reinforced posts improve the prognosis of endodontically treated teeth by restoring structural integrity and distributing functional stress. Their retention depends on the bond strength between the post, resin cement, and root dentin. Adhesive systems, such as total-etch and self-etch, play a key role in enhancing this bond, interacting differently with the dentin substrate [6].

Recent research has investigated dimethyl sulfoxide (DMSO), a strong organic solvent and penetration enhancer, as a dentin pretreatment agent. Pashley et al. evaluated the effect of DMSO on dentin prior to adhesive application [7]. Owing to its amphiphilic nature, small molecular size [8], and superior tissue penetration ability [9], DMSO is being explored as a solvent in dental adhesives. This study aims to evaluate the effect of DMSO pretreatment on the push-out bond strength of fiber posts using total-etch and self-etch adhesives, potentially enhancing clinical outcomes in post-endodontic restoration.

## Materials and methods

Ethical approval

This study received ethical clearance from the Institutional Ethics Committee of Sri Ramachandra Institute of Higher Education and Research (approval CSP/23/JUL/131/594).

Sample size calculation

The sample size was determined using GPower software version 3.1.9.4. The effect size (d = 1.67) was estimated based on the mean and SD values (Control: 9.66 ± 1.21; Study: 11.35 ± 1.28) reported in a previous study [10], using Cohen’s d formula with pooled SD. The calculation indicated a large effect size, which was used for sample size estimation in GPower 3.1 (α = 0.05, power = 0.80). With a study power of 95% and an effect size of 1.67, 10 samples per group were calculated, yielding a total of 40 samples across four groups (n = 10 per group).

**Figure 1:**
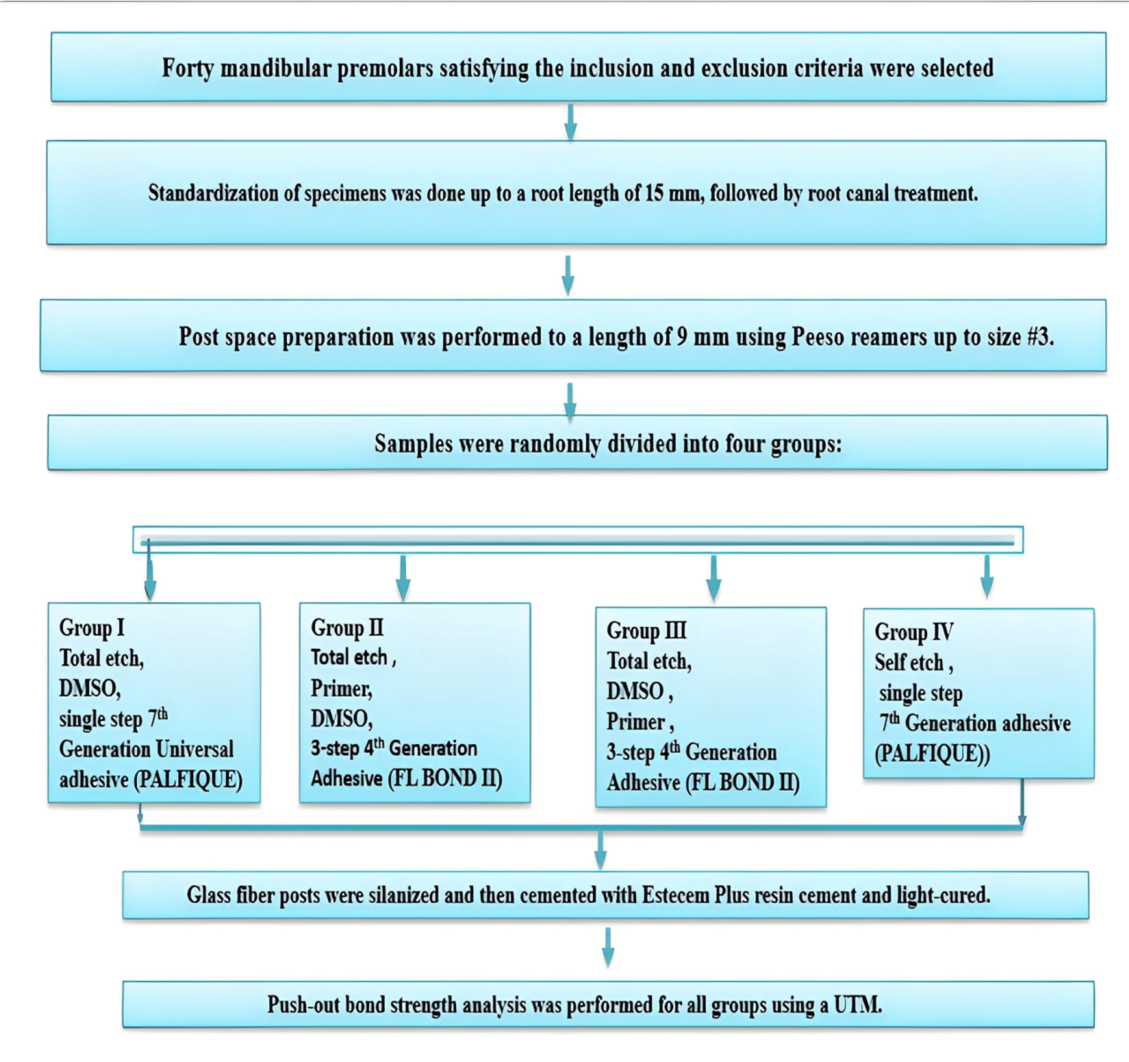
Flowchart depicting the step-by-step methodology for sample preparation, dentin pretreatment, bonding, luting of the fiber post, and push-out bond strength evaluation in the present study. DMSO, dimethyl sulfoxide; UTM, universal testing machine

The schematic representations of the experimental methodology are provided in Figure 1. Forty mandibular premolars satisfying the inclusion and exclusion criteria were selected.

Preparation of DMSO solution

**Figure 2:**
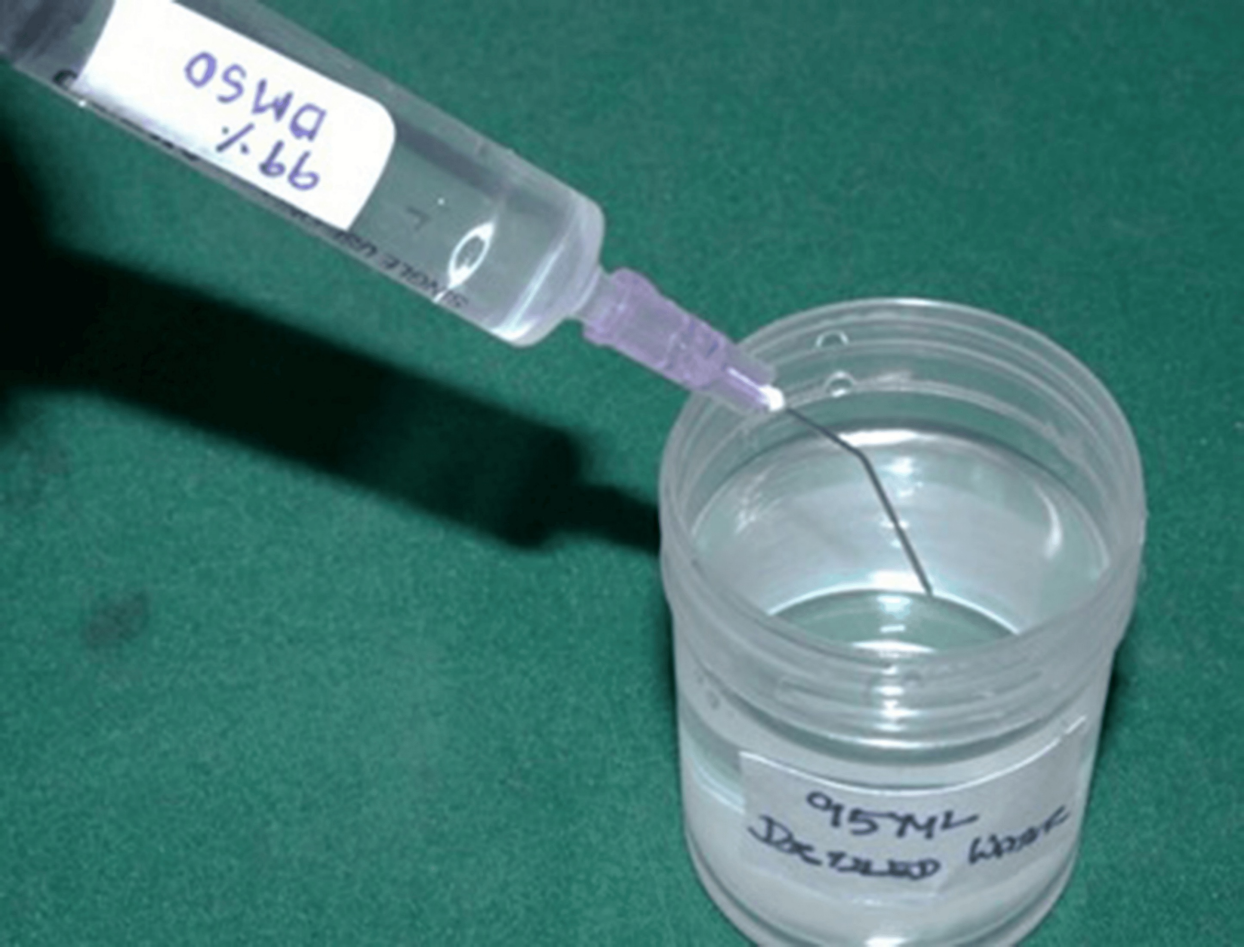
Preparation of 5% DMSO solution. DMSO, dimethyl sulfoxide

To prepare a 5% DMSO solution, 5 mL of 99.9% DMSO was measured and transferred into a clean container, followed by the addition of 95 mL of distilled water to obtain a final volume of 100 mL (Figure 2). The solution was gently mixed to ensure uniformity while minimizing foam formation [11].

Tooth selection and preparation

This in vitro experimental study was conducted in the Department of Conservative Dentistry and Endodontics, Sri Ramachandra Dental College and Hospital, Chennai, India. A total of 40 extracted human single-rooted premolar teeth, removed for orthodontic reasons, were collected from the Department of Oral and Maxillofacial Surgery, Sri Ramachandra Dental College (Chennai, India). Teeth exhibiting caries, fractures, or gross structural defects were excluded. To confirm the presence of a single canal, periapical radiographs were taken in both buccolingual and mesiodistal directions. Teeth identified with more than one canal were excluded from the study.

**Figure 3:**
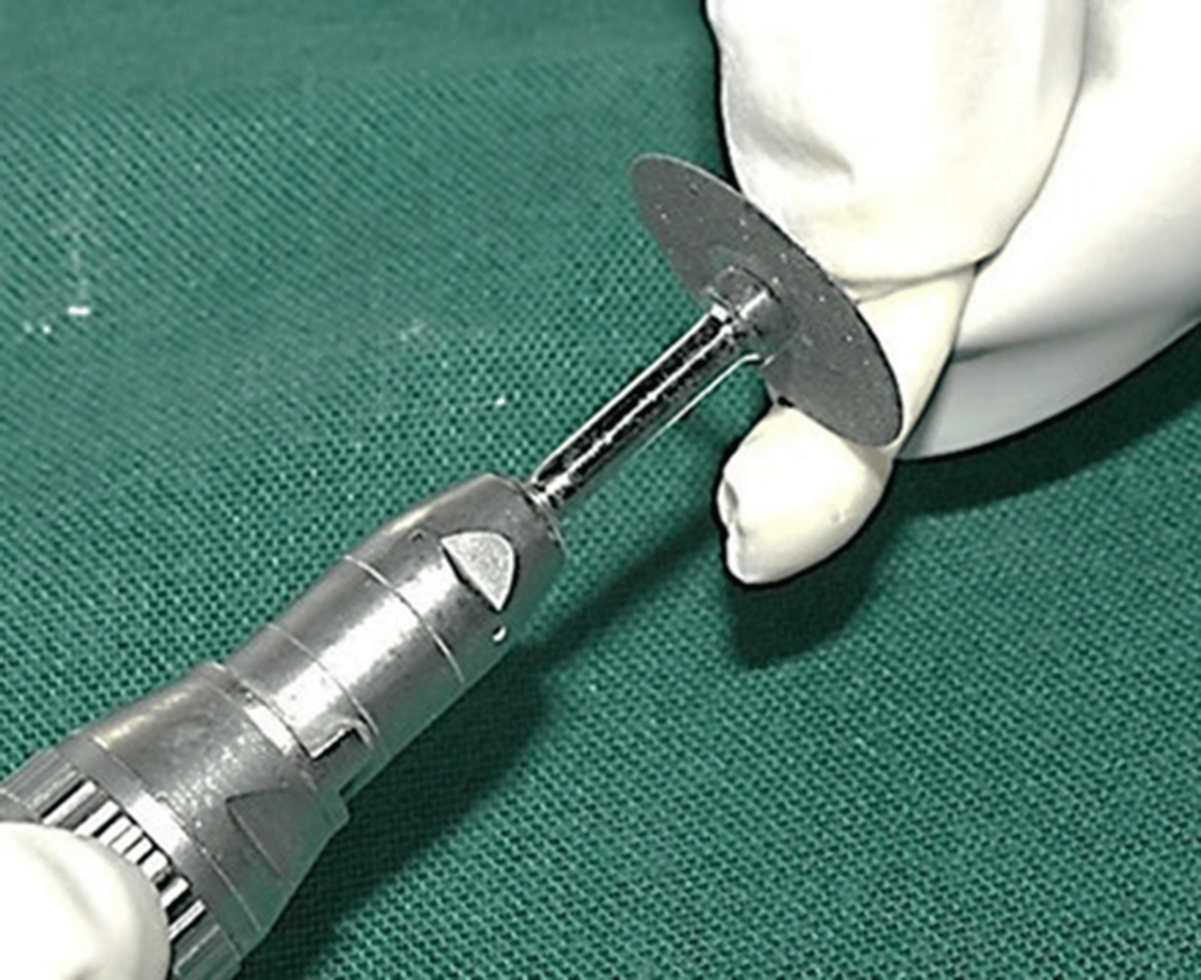
Sectioning of the sample to obtain a standardized length of 15 mm.

**Figure 4:**
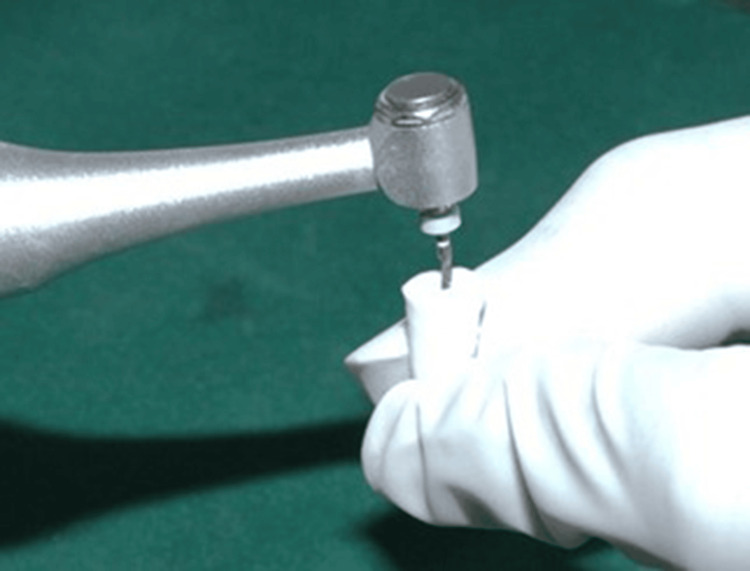
Instrumentation up to size F3 rotary file.

The crowns were sectioned using a diamond disc to obtain a standardized root length of 15 mm (Figure 3). The working length was measured with a size 15 K-file (Mani, INC., Utsunomiya, Japan) and verified radiographically by subtracting 1 mm from the actual canal length [12]. Cleaning and shaping were performed using ProTaper Gold rotary files (Dentsply Sirona, Baden, Switzerland) up to size F3 following the crown-down technique (Figure 4). Irrigation was performed with 1 mL of 5.25% NaOCl (Prime Dental Products Pvt Ltd, Thane, India) between each instrument. After final instrumentation, a 17% EDTA rinse was applied for one minute, followed by drying with absorbent paper points.

**Figure 5:**
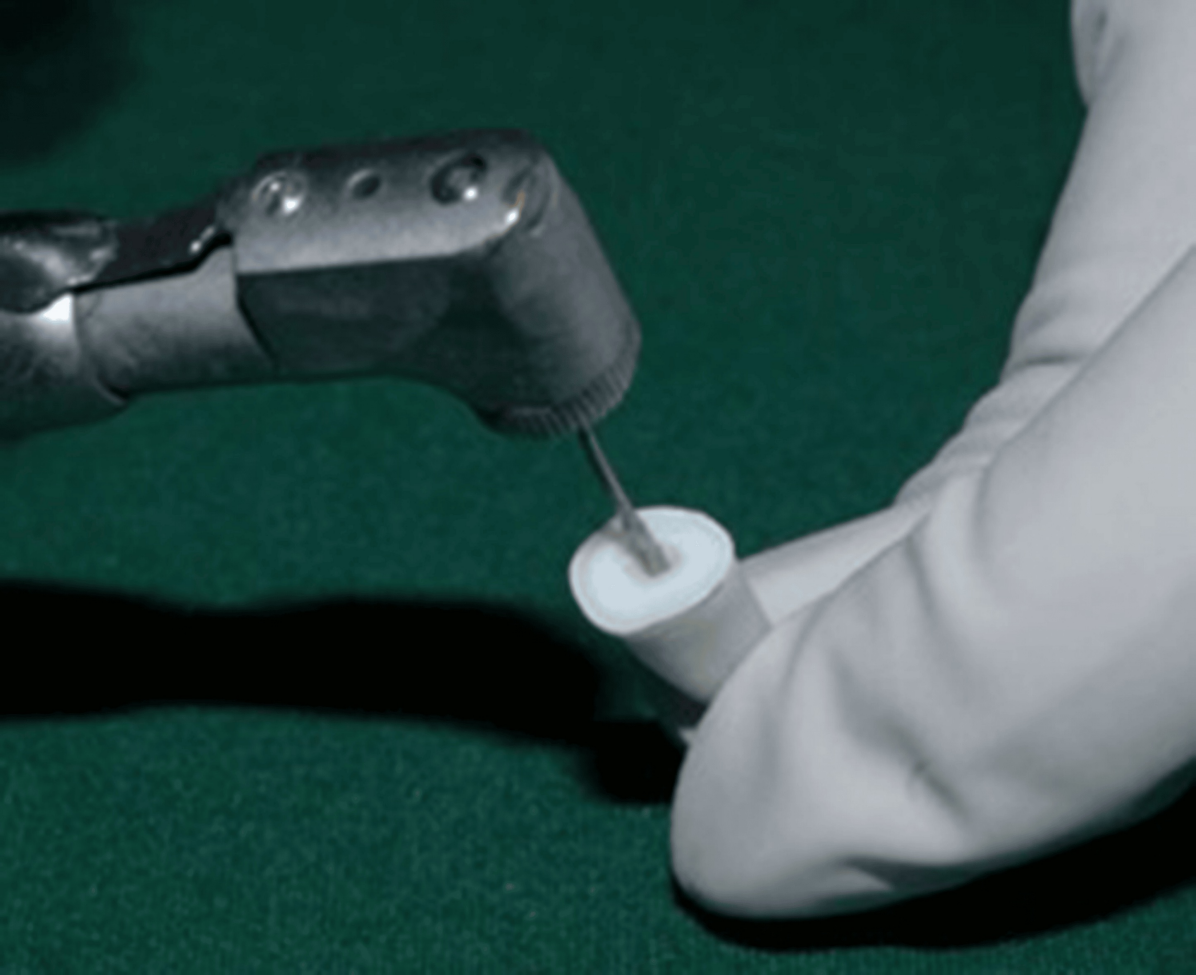
Post space preparation performed after obturation using Peeso reamers up to size 3.

Root canals were obturated using the single-cone technique with F3 gutta-percha cones (DiaDent, Cheongju-si, South Korea) and AH Plus sealer (Dentsply, Charlotte, NC, USA). Post space preparation was carried out using Peeso reamers (sizes 1 to 3, Mani INC.) to a depth of 9 mm, maintaining a 5 mm apical plug (Figure 5).

Study design

Following post space preparation, 40 standardized teeth were randomly assigned into four groups (n = 10), each receiving a different protocol for bonding the fiber post into the prepared root canal space. The variation among the groups was primarily based on the sequence and combination of etching, DMSO application, primers/adhesives, and the cementation of fiber posts. The groups were as follows: Group I: Total-etch + DMSO + seventh-generation universal adhesive (Palfique, Tokuyama Dental Corp., Tokyo, Japan); Group II: Total-etch + primer + DMSO + three-step, fourth-generation adhesive (FL Bond II System, Shofu Inc., Kyoto, Japan); Group III: Total-etch + DMSO + primer + three-step, fourth-generation adhesive (FL Bond II, Shofu Inc.); and Group IV: Self-etch + DMSO + seventh-generation universal adhesive (Palfique, Tokuyama Dental Corp.).

Group I

**Figure 6:**
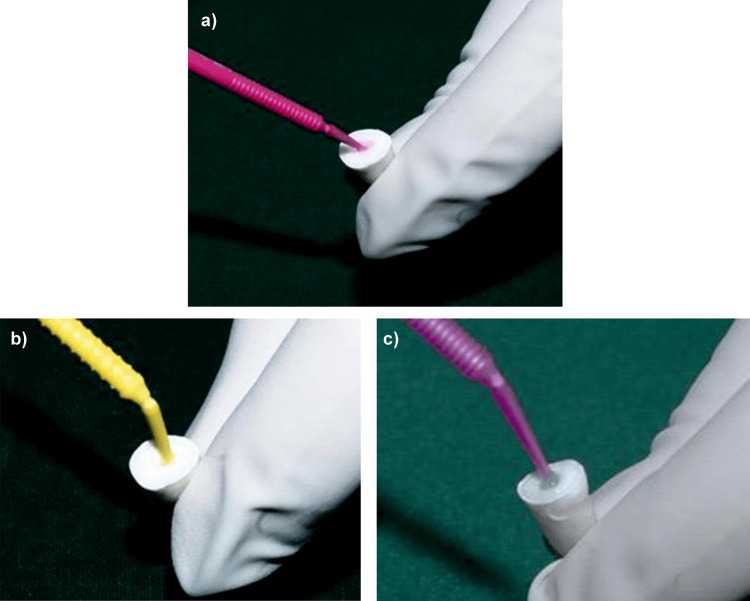
Group I: (a) Application of 37% phosphoric acid. (b) Application of DMSO solution. (c) Application of Palfique bond.

The post space was etched using 37% phosphoric acid (Prime Dental Products) for 15 seconds with microapplicator tips, then thoroughly rinsed with deionized water using a syringe and blot-dried with paper points. Freshly prepared DMSO (Pristine Potions, Rohtak, India) was gently applied to the canal walls with microapplicator tips for 60 seconds [9], followed by gentle blot drying with paper points to remove excess solution. The universal adhesive (Palfique Bond, Tokuyama Dental Corp.) was then applied to the canal walls using microapplicator tips, blot-dried with paper points, and light-cured for 20 seconds using an LED light-curing device (Figure 6).

Group II

**Figure 7:**
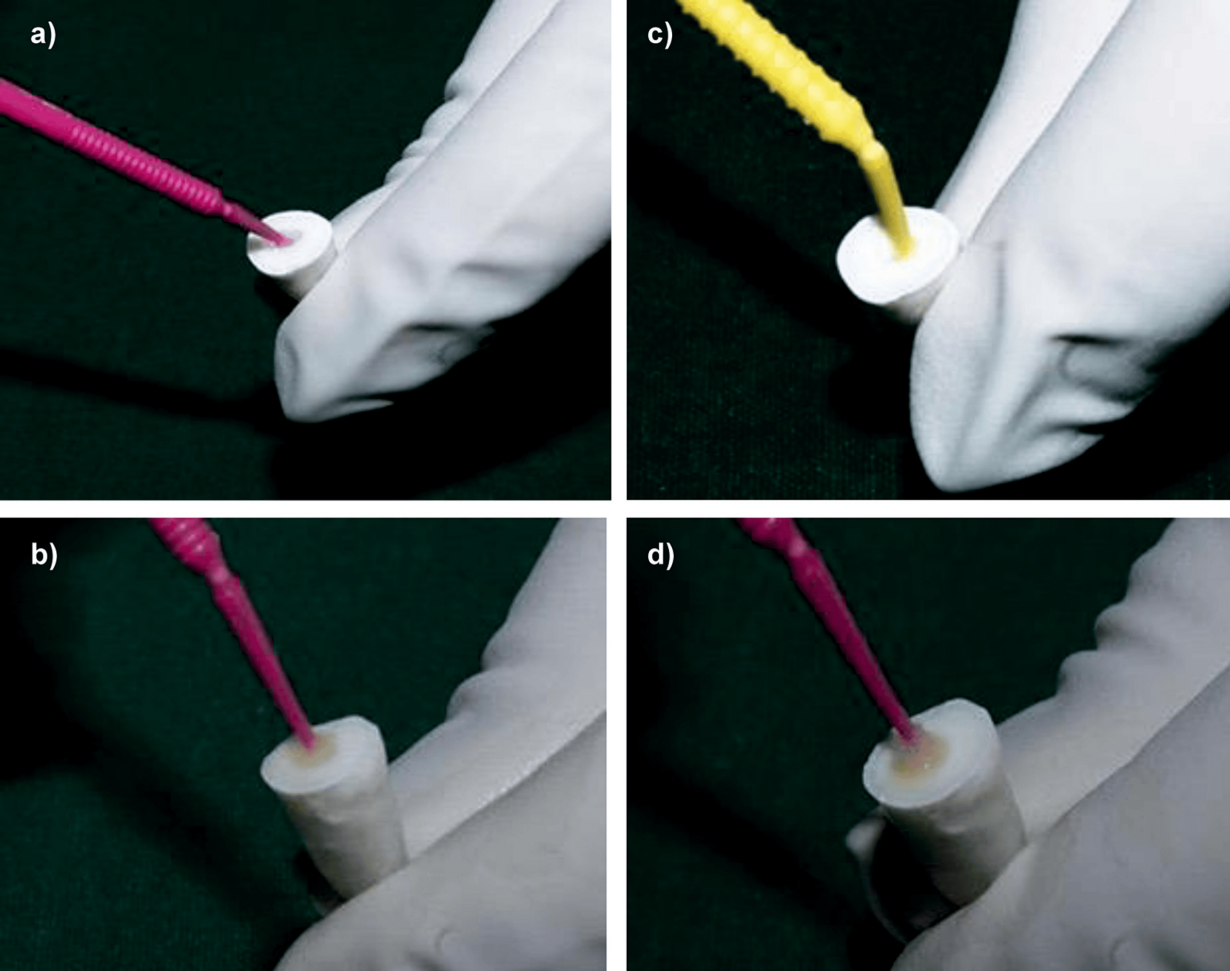
Group II: (a) Application of 37% phosphoric acid. (b) Application of DMSO solution. (c) Application of primer (FL Bond II). (d) Application of bonding agent (FL Bond II). DMSO, dimethyl sulfoxide

The post space was etched with 37% phosphoric acid for 15 seconds using microapplicator tips, thoroughly rinsed with water using a syringe, and blot-dried with paper points. The primer component of FL Bond II (Shofu Inc.) was applied for 10 seconds to the etched dentin surfaces using microapplicator tips, followed by blot drying with paper points to remove excess. After primer application, DMSO was applied for 60 seconds using microapplicator tips and gently blot-dried with paper points. The adhesive component of FL Bond II was then applied with microapplicator tips for 10 seconds and light-cured for 20 seconds using an LED light-curing device (Figure 7).

Group III

**Figure 8:**
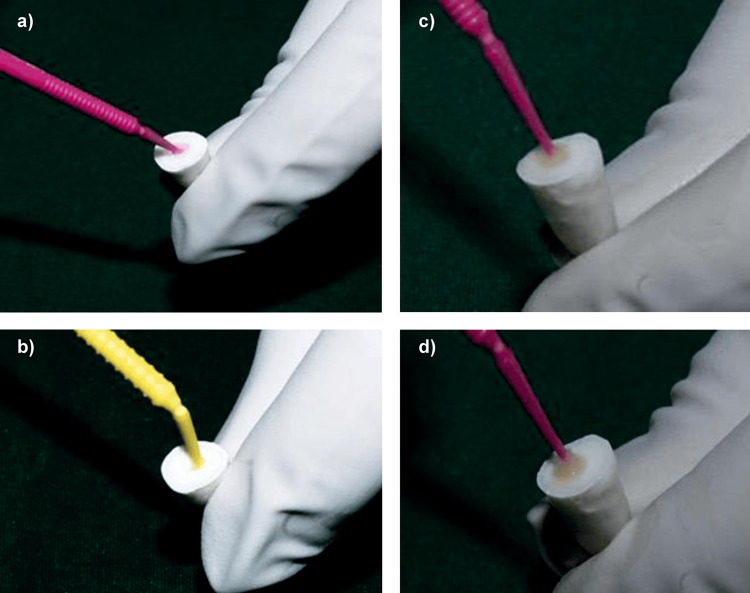
Group III: (a) Application of 37% phosphoric acid. (b) Application of primer (FL Bond II). (c) Application of DMSO solution. (d) Application of bonding agent (FL Bond II). DMSO, dimethyl sulfoxide

The post space was etched for 15 seconds using 37% phosphoric acid with microapplicator tips, rinsed thoroughly with water using a syringe, and blot-dried with paper points. DMSO was applied to the canal walls for 60 seconds using microapplicator tips and gently blot-dried with paper points. This was followed by the application of the primer component of FL Bond II for 10 seconds using microapplicator tips, then blot-dried with paper points to remove excess. The adhesive component was applied for 10 seconds and light-cured for 20 seconds using an LED light-curing device (Figure 8).

Group IV

**Figure 9:**
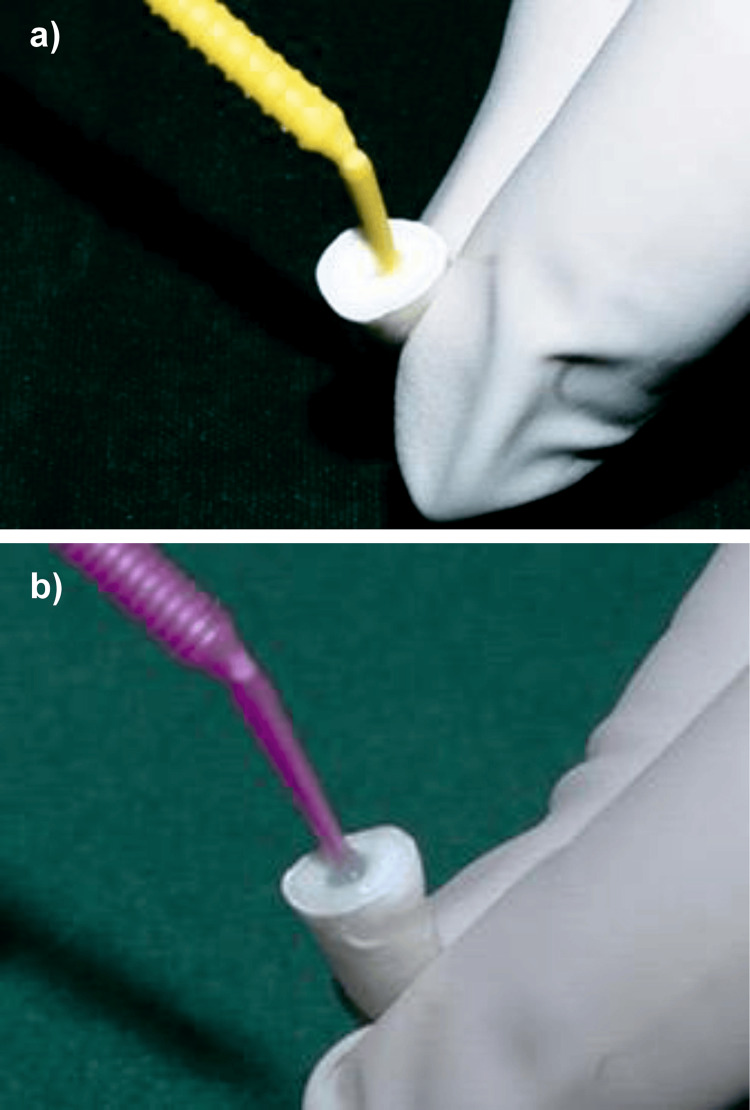
Group IV: (a) Application of DMSO solution. (b) Application of Palfique bond. DMSO, dimethyl sulfoxide

In this group, no prior acid etching was performed. DMSO was directly applied to the post space for 60 seconds using microapplicator tips and blot-dried with paper points. The universal adhesive (Palfique Bond, Tokuyama Dental Corp.) was then applied with microapplicator tips, gently blot-dried with paper points, and light-cured for 20 seconds using an LED light-curing device (Figure 9).

**Figure 10:**
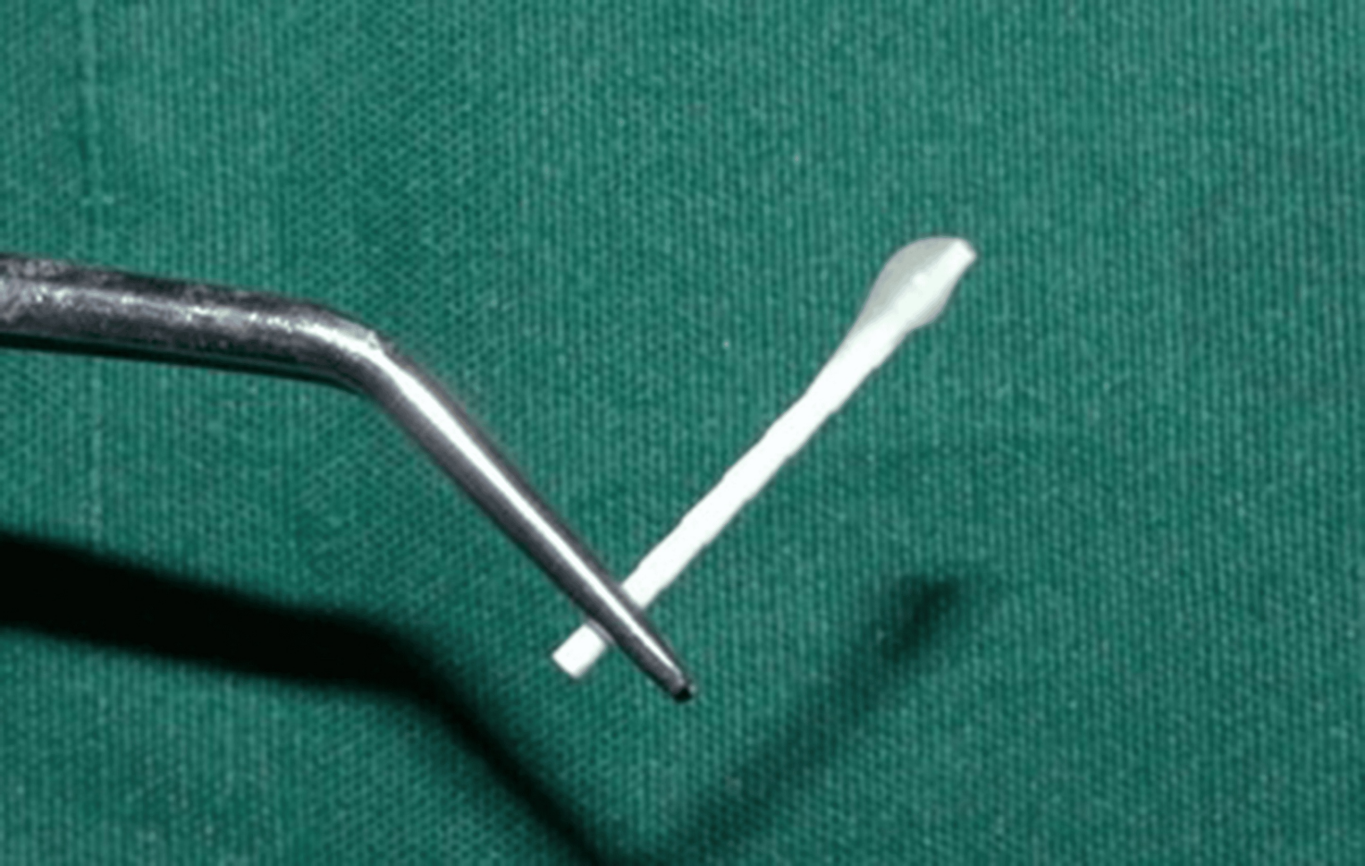
Coating of the post with resin cement.

**Figure 11:**
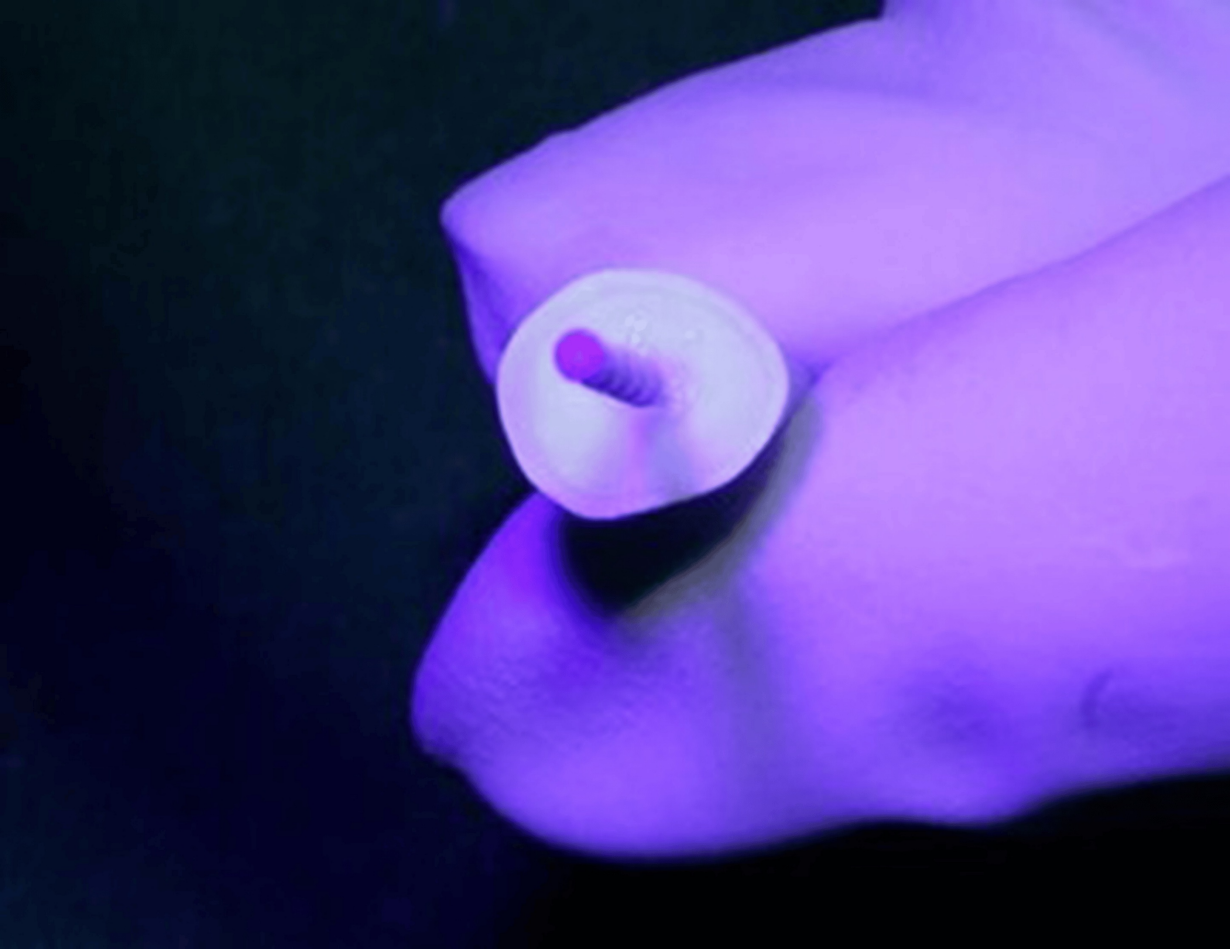
Fiber posts cemented and light-cured.

For all 40 samples, parallel-sided size 2 fiber posts (Angelus, Londrina, Brazil) were silanized with Silano Angelus (Angelus) and luted using Estecem Plus resin cement (Tokuyama Dental Corp.) (Figure 10). The cemented posts were then light-cured for 20 seconds (Figure 11).

Push-out bond strength evaluation

**Figure 12:**
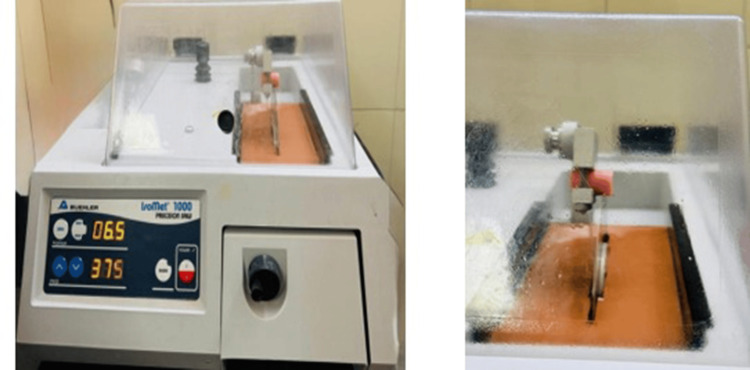
Sectioning of samples using Isomet 1000: (a) Isomet 1000 apparatus. (b) Sectioning of the samples.

**Figure 13:**
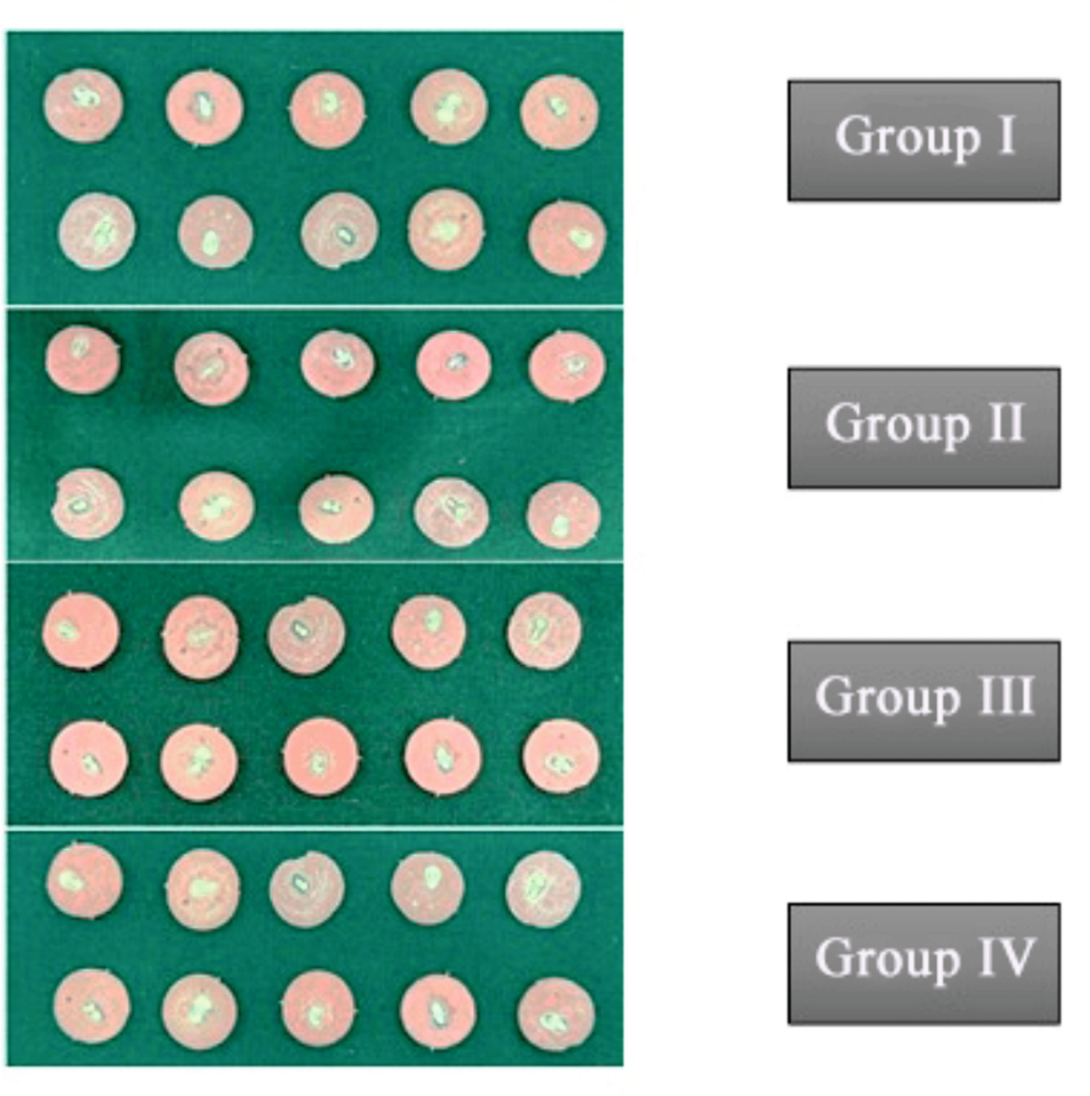
Sectioned samples prepared for evaluating push-out bond strength.

**Figure 14:**
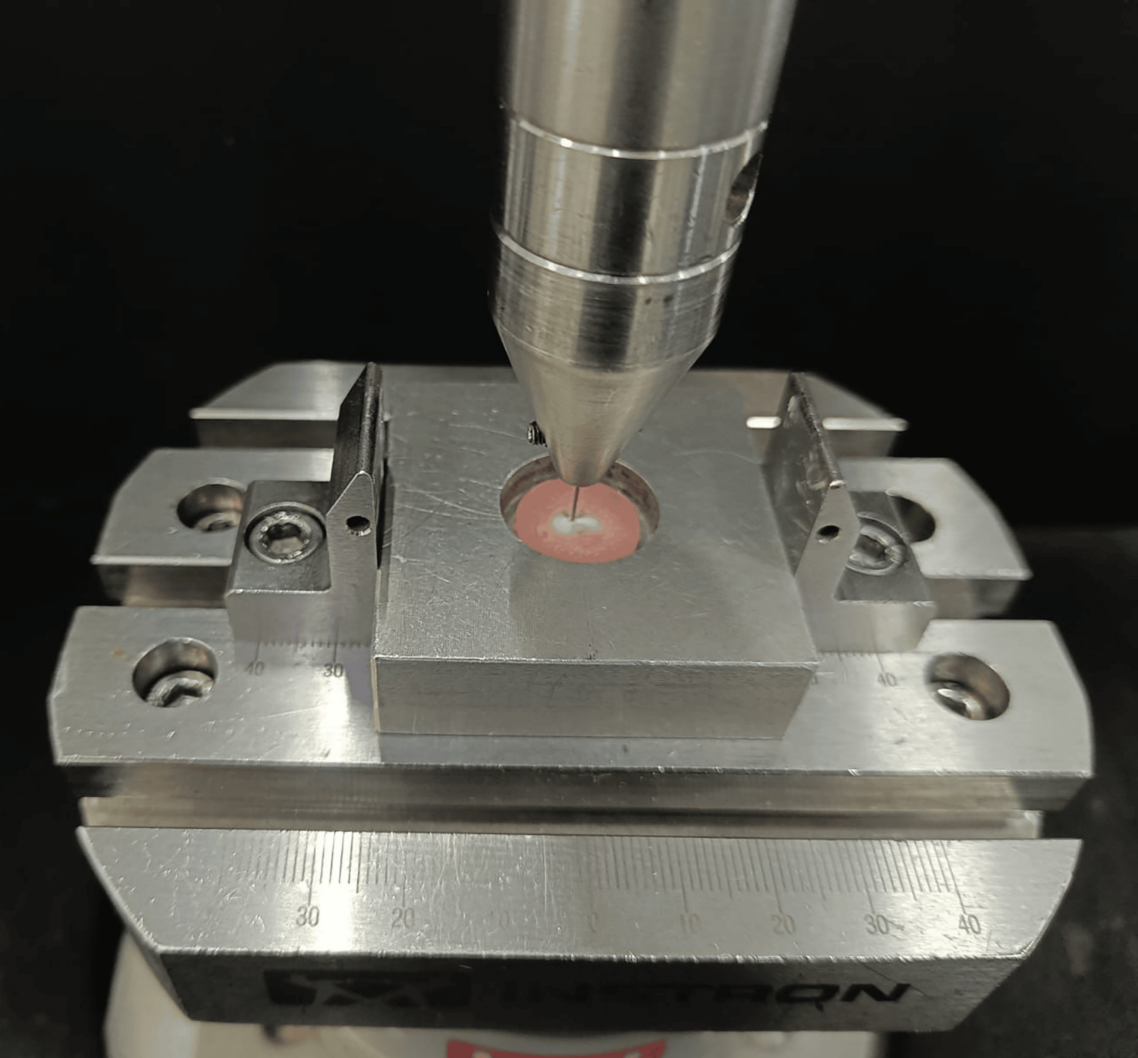
Push-out bond strength analysis using a UTM. UTM, universal testing machine

All samples were embedded in acrylic resin blocks and sectioned into 200 μm-thick discs using a hard tissue microtome under continuous water cooling (Figure 12). Forty sectioned samples were obtained for testing (Figure 13). Push-out bond strength was measured using a universal testing machine, applying force in an apical-to-coronal direction at a crosshead speed of 0.5 mm/min. A 0.5 mm diameter stainless steel plunger was centered on the post-tooth interface during testing (Figure 14).

The push-out bond strength (MPa) was calculated using the following formula:

$\text{Bond strength (MPa)} = \frac{\text{Force to dislodge (N)}}{\text{Surface area (mm}^2\text{)}}$

Operator calibration and reliability

All procedures were performed by a single calibrated operator (third-year postgraduate student). Calibration and training were conducted on pilot samples before the main experiment to ensure procedural uniformity. Intra-examiner reliability for push-out testing was verified by repeating measurements on randomly selected samples.

Statistical analysis

Statistical analysis was performed using IBM SPSS Statistics for Windows, Version 25.0 (Released 2017; IBM Corp., Armonk, NY, USA). The Kolmogorov-Smirnov test was used to assess the normality of numerical data, and the Shapiro-Wilk test was used to evaluate the homogeneity of variance. Results were expressed as mean ± SD. Data were analyzed at a 95% CI, with statistical significance set at p < 0.05. Intergroup comparisons were performed using one-way ANOVA, and pairwise comparisons were conducted using Tukey’s post hoc test. A p-value ≤ 0.05 was considered statistically significant.

## Results

To assess whether the collected data followed a normal distribution, both the Kolmogorov-Smirnov test and the Shapiro-Wilk test were applied to the dataset across all four experimental groups. The results of both normality tests indicated that the data were normally distributed (p > 0.05) (Table 1).

**Table 1:** Assessment of data normality using Kolmogorov-Smirnov and Shapiro-Wilk tests. The normality of data distribution was assessed using the Kolmogorov-Smirnov and Shapiro-Wilk tests. A significance level of α = 0.05 was adopted for all statistical analyses, with ^*^ p > 0.05 indicating no significant deviation from normality and supporting the use of parametric tests.

Group	Kolmogorov-Smirnov			Shapiro-Wilk		
	Statistic	Degree of freedom	p-Value	Statistic	Degree of freedom	p-Value
Group I	0.16	10	0.20^*^	0.93	10	0.44
Group II	0.17	10	0.20^*^	0.9	10	0.26
Group III	0.17	10	0.20^*^	0.89	10	0.17
Group IV	0.12	10	0.20^*^	0.97	10	0.91

One-way ANOVA was used to compare a quantitative dependent variable across multiple categorical groups, such as different skeletal patterns or clinical conditions. The descriptive statistics of one-way ANOVA showed that Group II had the highest mean bond strength (Total etch + Primer + DMSO + 2-step 4th Generation Adhesive (FL Bond II System)) (Table 2).

**Table 2:** One-way ANOVA for intergroup comparison of mean push-out bond strength. Mean values, SDs, and 95% CIs for each group are presented. The ANOVA test statistic (F) and corresponding p-value are also reported. An asterisk (^*^) indicates a statistically significant difference between groups (p < 0.05).

Group	Mean push-out bond strength (MPa)	SD	95% CI for mean		F	p-Value
			Lower bound	Upper bound		
Group I	0.68	0.31	0.45	0.9	15.3	<0.001^*^
Group II	2.34	0.5	1.98	2.69		
Group III	2.24	0.76	1.7	2.79		
Group IV	1.39	0.82	0.81	1.98		

The post hoc analysis highlighted the specific pairs of groups that showed statistically significant differences, as indicated by an asterisk (^*^). Statistically significant differences in bond strength were observed between Group II and Group IV, Group III and Group IV, Group I and Group II, and Group I and Group III. No significant differences were found between Group II and Group III or between Group I and Group IV (both involving Palfique universal adhesive with and without etching) (Table 3).

**Table 3:** Pairwise comparison of group mean push-out bond strength using Tukey’s post hoc test. Mean differences and associated pairwise p-values were computed using Tukey’s post hoc test. Statistical significance was set at p < 0.05. An asterisk (^*^) indicates a statistically significant difference.

Group	Group compared with	Mean difference (MPa)	p-Value
Group I	Group II	-1.66	<0.001^*^
	Group III	-1.57	<0.001^*^
	Group IV	-0.72	0.071
Group II	Group III	0.09	0.98
	Group IV	0.94	0.01^*^
Group III	Group IV	0.85	0.02^*^

## Discussion

Endodontic access preparation in teeth with loss of marginal ridges compromises structural integrity, increasing the risk of failure. Nagpal et al. suggested that while endodontic treatment minimally affects tooth biomechanics, the strength of a tooth is directly related to the amount of coronal tissue lost due to caries or restorations [13]. In cases of extensive tissue loss from caries or trauma, fiber-reinforced composite (FRC) resin posts are commonly used to retain the core material.

FRC posts are preferred because their modulus of elasticity is similar to that of dentin, promoting uniform stress distribution into the radicular dentin and reducing stress concentration at the bonding interface [14]. Both clinical and in vitro studies have shown that FRC posts reduce the risk of vertical root fracture [15], supporting their use in this study. However, bonding FRC posts to root canal dentin remains challenging due to variable adhesion outcomes.

The cementation protocol involves post surface preparation and dentin conditioning using adhesives. The Angelus Reforpost used in this study is non-silanated. Silanization with Silano Angelus enhances chemical bonding by forming covalent bonds between quartz fibers and resin. Pai et al. demonstrated that dual-cure resin cements are preferred for luting because of their superior flexural strength and higher degree of monomer conversion [16]. Therefore, Estecem Plus dual-cure resin cement was selected for this study.

Debonding at the resin-dentin interface often occurs due to incomplete hybridization within the confined root canal space. Traditionally, 3-step etch-and-rinse adhesives are favored for fiber post cementation because they effectively remove the thick smear layer produced during post space preparation [17]. Among these, FL Bond II, a fourth-generation adhesive containing 10-MDP monomer, shows high bond strength. MDP forms a stable calcium-phosphate-MDP salt, contributing to long-term adhesion. Additionally, Mehmood et al. showed that ethanol-wet bonding following DMSO pretreatment enhances bond durability [18]. Since FL Bond II contains 10-MDP and ethanol, it was used as the total-etch adhesive in this study.

Although effective, etch-and-rinse adhesives are technique-sensitive and time-consuming, leading to the development of universal adhesives. Universal adhesives are versatile and can be used in both self-etch and etch-and-rinse modes. Two-bottle self-cured universal adhesives offer improved bond stability because polymerization begins after mixing, enabling three-dimensional cross-linking with calcium and SR monomers [19]. Rai and Naik found that self-cured universal adhesives yield the highest bond strength [20]. Therefore, Palfique, a self-cured two-bottle universal adhesive, was used in this study.

DMSO has recently gained attention as a dentin pretreatment agent due to its ability to disrupt collagen cross-linking and increase dentin wettability. As a dipolar aprotic solvent with amphiphilic properties, DMSO enhances adhesive infiltration [21]. Tjäderhane et al. demonstrated that 5% DMSO improves bond strength by increasing wettability and preserving collagen [22,23]. Based on these findings, 5% DMSO was used in this study.

Push-out bond strength testing was selected because it distributes stress uniformly and reduces premature failures, producing reliable data. This study aimed to determine the optimal DMSO application protocol for FRC post cementation using the following groups: Group I: Total-etch + DMSO + 7th Generation Universal Adhesive (Palfique); Group II: Total-etch + Primer + DMSO + 3-step 4th Generation Adhesive (FL Bond II); Group III: Total-etch + DMSO + Primer + 3-step 4th Generation Adhesive (FL Bond II); and Group IV: Self-etch + DMSO + 7th Generation Universal Adhesive (Palfique).

Group II exhibited the highest bond strength. Statistically significant differences were found between Group II and Group IV, Group III and Group IV, Group I and Group II, and Group I and Group III. The superior performance of the total-etch groups can be attributed to 35-37% phosphoric acid, which removes the smear layer and exposes collagen fibrils for improved resin tag formation and micromechanical bonding.

DMSO likely enhanced bonding by disrupting collagen cross-links, increasing collagen fibril spacing, reducing hydrolytic enzyme activity, and improving resin infiltration [24]. It enhances adhesive spread and encapsulation of collagen while minimizing trapped water, which improves resin tag stability and reduces hydrolytic degradation [25]. Interestingly, no significant difference was observed between Group II and Group III, indicating that the sequence of DMSO and primer application did not influence bond strength.

In universal adhesives, DMSO had limited effect. Palfique, with a pH of 2.8, can etch dentin similarly to phosphoric acid, releasing calcium ions that react to form calcium phosphate (DCPM or brushite). In self-etch mode, these precipitates remain on the dentin surface, interfering with monomer infiltration and reducing micromechanical retention. DMSO could not overcome this interference [26]. Therefore, Group IV (self-etch DMSO + Palfique) showed inferior bonding due to the lack of smear layer removal and the presence of mineral precipitates. In contrast, total-etch groups benefit from rinsing steps that eliminate such byproducts [19,27].

DMSO pretreatment had limited influence on universal adhesive groups, regardless of etching mode. Being a polar aprotic solvent with strong water affinity, DMSO can retain moisture on dentin surfaces, particularly in universal adhesives where hydrophilic and hydrophobic components are applied together [28]. This residual moisture can hinder solvent evaporation and interfere with bond formation [29]. Even with extended application, DMSO may still reduce bond strength due to incomplete evaporation [30].

Yamauchi et al. reported similar bonding outcomes in universal adhesives regardless of etching method [31], which aligns with the present findings. Thus, universal adhesive groups showed inferior bond strength in both modes, possibly due to DMSO-induced residual moisture and inadequate rinsing, reinforcing the superiority of total-etch protocols with DMSO and multistep adhesives.

A limitation of this study is that it was conducted in vitro and therefore cannot fully replicate the complex oral environment, where factors such as saliva, pH fluctuations, bacterial biofilm, and masticatory stress influence adhesive performance. Although thermal cycling can partially simulate intraoral conditions, it still does not completely mimic clinical reality. Furthermore, the study was short-term; thus, the long-term durability of resin-dentin bonds and clinical post retention were not assessed.

Clinically, DMSO pretreatment in combination with total-etch, multistep adhesives significantly improves the bonding of FRC posts to root dentin. This approach may reduce debonding and root fractures, thereby enhancing the long-term success of post-retained restorations.

## Conclusions

The application of 5% DMSO as a dentin pretreatment agent influenced the push-out bond strength of fiber posts differently depending on the adhesive strategy employed. The highest bond strength was observed in Group II, where DMSO was applied after the primer in a two-step total-etch adhesive system (FL Bond II), suggesting a synergistic effect between the primer and DMSO in enhancing resin infiltration and hybrid layer formation. Total-etch techniques, particularly those involving separate primer and adhesive applications, outperformed self-etch and universal adhesive approaches. Conversely, the application of DMSO in self-etch protocols did not yield significant improvement, likely due to limited collagen exposure and interference with adhesive monomer infiltration. These findings support the use of DMSO as a beneficial pretreatment in total-etch adhesive protocols, especially when used with a separate primer system, but highlight its limited effectiveness in self-etch or universal adhesive strategies.
